# Evaluation of the broth microdilution plate methodology for susceptibility testing of *Mycobacterium tuberculosis* in Peru

**DOI:** 10.1186/s12879-022-07677-9

**Published:** 2022-08-24

**Authors:** Zully M. Puyén, David Santos-Lázaro, Aiko N. Vigo, Jorge Coronel, Miriam J. Alarcón, Vidia V. Cotrina, David A. J. Moore

**Affiliations:** 1 Instituto Nacional de Salud, Lima, Perú; 2 Escuela de Medicina, Universidad Peruana de Ciencias Aplicadas, Lima, Perú; 3 Universidad Peruana Cayetano Heredia, Lima, Perú; 4 London School of Hygiene & Tropical Medicine, London, UK

**Keywords:** Mycobacterium tuberculosis, Drug susceptibility testing, Drug resistance, Antimicrobial agents, Minimum inhibitory concentration, Broth microdilution

## Abstract

**Background:**

Tuberculosis (TB) is a communicable, preventable and curable disease caused by the bacterium *Mycobacterium tuberculosis* (MTB). Peru is amongst the 30 countries with the highest burden of multidrug-resistant tuberculosis (MDR-TB) worldwide. In the fight against drug-resistant tuberculosis, the UKMYC6 microdilution plate was developed and validated by the CRyPTIC project. The objective of the study was to evaluate the use of the broth microdilution (BMD) plate methodology for susceptibility testing of drug-resistant MTB strains in Peru.

**Methods:**

MTB strains isolated between 2015 and 2018 in Peru were used. 496 nationally-representative strains determined as drug-resistant by the routine 7H10 Agar Proportion Method (APM) were included in the present study. The Minimum Inhibitory Concentration (MIC) of 13 antituberculosis drugs were determined for each strain using the UKMYC6 microdilution plates. Diagnostic agreement between APM and BMD plate methodology was determined for rifampicin, isoniazid, ethambutol, ethionamide, kanamycin and levofloxacin. Phenotypes were set using binary (or ternary) classification based on Epidemiological cut-off values (ECOFF/ECV) proposed by the CRyPTIC project. Whole Genome Sequencing (WGS) was performed on strains with discrepant results between both methods.

**Results:**

MIC distributions were determined for 13 first- and second-line anti-TB drugs, including new (bedaquiline, delamanid) and repurposed (clofazimine, linezolid) agents. MIC results were available for 80% (397/496) of the strains at 14 days and the remainder at 21 days. The comparative analysis determined a good agreement (0.64 ≤ k ≤ 0.79) for the drugs rifampicin, ethambutol, ethionamide and kanamycin, and the best agreement (k > 0.8) for isoniazid and levofloxacin. Overall, 12% of MIC values were above the UKMYC6 plate dilution ranges, most notably for the drugs rifampicin and rifabutin. No strain presented MICs higher than the ECOFF/ECV values for the new or repurposed drugs. Discrepant analysis using genotypic susceptibility testing by WGS supported half of the results obtained by APM (52%, 93/179) and half of those obtained by BMD plate methodology (48%, 86/179).

**Conclusions:**

The BMD methodology using the UKMYC6 plate allows the complete susceptibility characterization, through the determination of MICs, of drug-resistant MTB strains in Peru. This methodology shows good diagnostic performances for rifampicin, isoniazid, ethambutol, ethionamide, kanamycin and levofloxacin. It also allows for the characterization of MICs for other drugs used in previous years against tuberculosis, as well as for new and repurposed drugs recently introduced worldwide.

**Supplementary Information:**

The online version contains supplementary material available at 10.1186/s12879-022-07677-9.

## Background

Tuberculosis (TB) is a communicable, preventable and curable disease caused by the bacterium *Mycobacterium tuberculosis* (MTB). It is the 13th leading cause of death and the second leading infectious killer after COVID-19 (ranking above HIV/AIDS) [1]. It is estimated that in 2019, TB was diagnosed in 10 million (range 8.9–11 million) people and 1.4 million died worldwide from this disease [2]. The problem of managing and eliminating TB is further exacerbated by the presence of drug-resistant TB, a major public health problem that threatens progress made in TB care and control worldwide [3]. In 2019, about half a million people developed rifampicin-resistant TB, of which 78% were multidrug-resistant TB (MDR-TB) [2]. Also, in 2018 it was estimated that 6.2% of MDR-TB cases were extensively drug-resistant TB (XDR-TB) [3].

Peru has 14% of the estimated cases of tuberculosis in the Region of the Americas, with 27,000 new cases of active disease and 17,000 new cases of smear-positive pulmonary TB each year. In addition, it is one of the 30 countries in the world with the highest burden of MDR-TB [3]. TB and MDR-TB are distributed in the 24 Departments of Peru; however, the department of Lima (capital of Peru) and the constitutional province of Callao account for 61% of TB cases and 78% of MDR-TB and XDR-TB cases [4].

Different methodologies have been implemented over the years in Peru for the evaluation of resistance to the drugs used in the treatment of tuberculosis. Since 2004 the gold standard method at the National Mycobacterial Reference Laboratory for *Mycobacterium tuberculosis* complex susceptibility testing has been the 1% indirect 7H10 Agar Proportion Method (APM), which is laborious and requires 2–3 weeks from strain inoculation for results to become available [5].

The CRyPTIC (Comprehensive Resistance Prediction for Tuberculosis: An International Consortium) research project has validated the UKMYC6 broth microdilution (BMD) plate to provide the simultaneous evaluation of Minimum Inhibitory Concentration (MIC) of several anti-tuberculosis drugs from a single clinical isolate of MTB. UKMYC6 plate is a variant of the original MYCOTB plate [6, 7] and contains 13 different anti-TB drugs, including two repurposed (linezolid and clofazimine) and two new (bedaquiline and delamanid) compounds [8]. The original MYCOTB microdilution plate showed good results of categorical agreement (92–100%) for the determination of susceptibility to the conventional first- and second-line drugs [6, 7] evaluated by the APM; however, it was only with the development of the UKMYC5 plate that the incorporation of new and repurposed drugs was achieved [8]. During preliminary evaluation of this plate the drug para-aminosalicylic acid was eliminated and concentration ranges for the remaining drugs were optimized, giving rise to the UKMYC6 plate. In this way, the UKMYC6 microdilution plate provides quantitative MIC values and thus a higher resolution understanding of drug resistance, potentially facilitating improved, individualized treatment for each patient.

The objective of this study was to take advantage of the opportunity presented by the CRyPTIC study of genomic determinants of drug resistance to evaluate the performance of the BMD methodology using UKMYC6 plate for susceptibility testing of MTB strains to antituberculosis drugs in Peru when compared with the APM results. Furthermore, through selection of a nationally representative sample of MTB strains, the profile of TB drug MICs in drug-resistant strains nationally can be elucidated.

## Methods

### Design, settings and selection of MTB strains

The study was carried out by the National Reference Laboratory for Mycobacteria (LRNM [*Laboratorio de Referencia Nacional de Micobacterias*]) of the National Institute of Health (INS [*Instituto Nacional de Salud*]) in collaboration with investigators at Universidad Peruana Cayetano Heredia (UPCH) and the London School of Hygiene and Tropical Medicine (LSHTM). Drug-resistant MTB strains (according to routine APM results), representative from all over Peru were selected. Strains were randomly selected by stratified sampling according to the local burden of MDR-TB in each one of the 24 departments (in addition to the constitutional province of Callao) of Peru, reported in the 2015–2018 period [4]. Each of the selected strains were previously isolated from samples of patients with pulmonary and/or extrapulmonary TB during the mentioned period. All strains had preliminary information on resistance profiles obtained by the routine APM and were obtained from the LRNM culture bank.

### Routine susceptibility testing (APM testing)

The susceptibility tests were carried out under programmatic conditions by the LRNM in the period 2015–2018 using the APM. The procedures established by the Clinical and Laboratory Standards Institute (CLSI) [9] were followed and the phenotypic susceptibility was determined for the drugs rifampicin, isoniazid, ethambutol, ethionamide, kanamycin and levofloxacin, according to the critical concentrations (CC) recommended by the World Health Organization (WHO) at the date of testing (Table 1). Briefly, four quadrant Petri dishes containing Middlebrook 7H10 medium (Becton–Dickinson, Sparks, Md., USA) were used. MTB cultures on Lowenstein Jensen were transported to the LRNM from regional laboratories. The strains were sub-cultured in Middlebrook 7H9 broth (Becton Dickinson, Sparks, USA) and incubated for 7 days at 37 °C. Subsequently, fresh broth cultures were standardized to a McFarland 0.5 turbidity scale. The standardized suspensions were diluted 10^–2^ to allow the growth of countable colonies for interpretation. For each culture, the drug-containing quadrants, as well as the drug-free control quadrant, were inoculated with 100 µL of the diluted suspension. The plates were sealed in plastic bags and incubated at 37 °C for 21 days. The cultures were classified as resistant when the number of colonies developed in the drug quadrant was more than 1% of the number of colonies observed in the control quadrant, otherwise they were classified as susceptible.

**Table 1:** Drug concentrations values used for comparison between APM and BMD UKMYC6 plate methodology

Drug	APM CC (mg/L)	UKMYC6 ECOFF/ECV (mg/L)	UKMYC6 Borderline (mg/L)	UKMYC6 concentration ranges (mg/L)
Drugs used in drug-susceptible TB regimens				
Rifampicin	1.0	0.5	–	0.03–8
Rifabutin	–	0.12	–	0.06–2
Isoniazid	0.2	0.1	0.2, 0.4	0.025–12.8
Drugs used in MDR-TB regimens^a^				
Group A				
Moxifloxacin	–	1.0	–	0.06–4
Levofloxacin	1.0	1.0	–	0.125–8
Bedaquiline	–	0.25	–	0.008–1
Linezolid	–	1.0	–	0.06–4
Group B			–	
Clofazimine	–	0.25	–	0.03–2
Group C				
Ethambutol	5.0	4.0	4	0.25–32
Delamanid	–	0.12	–	0.008–0.5
Amikacin	–	1.0	–	0.25–16
Ethionamide	5.0	4.0	–	0.25–8
Additional				–
Kanamycin	5.0	4.0	–	1–16

^a^Groups A, B and C were set according to WHO update—2018 [10]

*APM*: 7H10 Agar Proportion Method, *BMD*: Broth Microdilution, *CC*: Critical Concentration, *ECOFF/ECV*: Epidemiological Cut-off Values

### Re-culture and susceptibility testing (BMD testing)

Drug susceptibility testing was performed using the UKMYC6 96-well microdilution plate format, designed by the CRyPTIC project (Thermo Fisher Inc., UK). The UKMYC6 plate allowed for the determination of susceptibility against 13 antituberculosis drugs composed of agents used in drug-susceptible TB treatment (rifampicin, rifabutin, isoniazid) as well as longer MDR-TB treatment corresponding to groups A (levofloxacin, moxifloxacin, bedaquiline, linezolid), B (clofazimine), C (ethambutol, delamanid, amikacin, ethionamide) and kanamycin (Table 1). Testing for each drug entailed between 5 and 10 concentrations obtained by serial doubling dilution (Additional file 1: Fig. S1). Previously cryopreserved strains were reactivated in 7H9 liquid medium, supplemented with “oleic acid albumin dextrose catalase” (OADC) (Thermo Fisher, Scientific Inc., USA), for 7 days at 37 °C. These were then subcultured in Middlebrook 7H10 media for 25–30 days at 37 °C. From the solid cultures in 7H10 medium, 0.5 McFarland scale suspensions were prepared in Tween saline with glass beads (Thermo Fisher, Scientific Inc., USA). Then, 100 µL of the suspension was diluted in a 7H9 broth tube supplemented with OADC to give an approximate inoculum of 1 × 10^5^ CFU/mL. Subsequently, using the automated Sensititre Autoinoculator^®^/AIM^®^ equipment (Thermo Fisher, Scientific Inc., USA), 100 µL of inoculum was dispensed into each well of the UKMYC6 plate. The plates were sealed using clear plastic and incubated aerobically at 35–37 °C. The H37Rv ATCC 27294 strain was used to perform periodic quality control tests of the analyzed drugs, as well as to quality control for contamination and adequate growth in two positive control (drug-free) wells of each plate used. All laboratory work related to the culture of live bacteria was carried out in the biosafety level 3 facilities of the INS and UPCH.

### Determination of MICs and susceptibilities

Plates were read using the semi-automated Vizion™ instrument. The results of the plates were considered valid only when the positive control wells showed acceptable growth and free of contamination. The plates were read 14 days after inoculation, as established by the CRyPTIC project [8]. Additionally, in accordance with the Standard Operating Procedure (SOP), if the growth of the positive control was weak or insufficient, a second reading was carried out at 21 days. The Vizion™ system captured and stored an image of the recorded MICs of each plate. The MIC of a drug was considered as the lowest concentration capable of inhibiting the visible growth of MTB in a given well. Based on the MICs, strains were categorized as susceptible, intermediate or resistant (ternary categorization) taking as reference the Epidemiologic Cut-off Values (ECOFF/ECV), as well as borderline concentrations (concentrations at which genetic mutations present different effects giving rise to mixed categories of susceptibility or resistance), established by the CRyPTIC project [11]. A result was determined as susceptible if the MIC was less than or equal to the established ECOFF/ECV; otherwise, it was defined as resistant. Isoniazid, ethambutol and ethionamide were categorized taking into account borderline concentrations (Additional file 1: Table S1).

### DNA extraction and genomic sequencing

WGS was performed for all strains for the primary objective of the parent trial. For this study, genomic results were only used for discrepant analysis to resolve discordant results between the APM and BMD tests. DNA extraction and BMD test were performed starting from the same solid culture (7H10 culture) to minimize phenotypic and genotypic variation. Genomic DNA was isolated using the phenol–chloroform method (Additional file 1: Supplementary Method). Sequencing libraries were generated using the Nextera XT Library Preparation Kit, following the manufacturer's recommendations. WGS was performed at the University of Oxford (UK) using the Illumina HiSeq platform (Illumina Inc., San Diego, CA, USA). Paired end 150 bp sequencing reads were generated and stored in fastq.gz files.

### Bioinformatic analysis

The quality of the sequencing reads was evaluated using the FastQC v0.11.9 [12] program. The paired reads were filtered with the Trimmomatic v0.38 [13] program using default values and a minimum Phred score of 20. The filtered reads were mapped against the H37Rv reference genome (NC_000962.3) using BWA v0.7.17 [14]. The elimination of duplicate readings was carried out with the program Picardtools v2.18.25 [15]. The variant call was made using the GATK v4 [16] program. Resistance-associated genes were evaluated for genotypic resistance to rifampin (*rpoB*), isoniazid (*inhA*, *katG*, *ahpC*, *fabG1*, *kasA*), fluoroquinolones (*gyrA*, *gyrB*), and second-line injectables (*rrs*, *eis*, *tlyA*). The genetic variants found were validated using the list of mutations published by the WHO in the “Catalogue of mutations in *Mycobacterium tuberculosis* complex and their association with drug resistance” [17]. Likewise, the resistance profiles were determined using the programs TBProfiler v3.0.4 [18] (database of mutations v. a2a234b) and Mykrobe v0.10 [19]. For each drug, the strains were classified as susceptible or resistant according to the absence or presence of mutations detected in the genes associated with resistance, respectively.

### Statistical analysis

Descriptive analyses of the MICs obtained and development times were performed on the UKMYC6 plates. For the six drugs evaluated by both APM and BMD method, diagnostic performance indices (sensitivity, specificity, positive predictive value, negative predictive value, categorical agreement and Cohen's Kappa coefficient) were determined by taking as reference values ​​the results of the APM and comparing them to the susceptible/resistant categorization from the BMD method (results with borderline MICs were excluded from analysis). All the calculations were performed in the program R v4.0.5 [20] using the packages epiR v2.0.19 (https://cran.r-project.org/web/packages/epiR) and vcd v1.4 (https://cran.r-project.org/web/packages/vcd). To determine the strength of statistical agreement, the kappa (k) value was used and was graded as insignificant (0 ≤ k ≤ 0.2), medium (0.2 < k ≤ 0.4), moderate (0.4 < k ≤ 0.6), good (0.6 < k ≤ 0.8) or almost perfect (0.8 < k ≤ 1) according to the previously proposed classification [21].

## Results

### Samples

The study analysed 496 MTB drug-resistant strains of which 70% (347/496) were from Lima and Callao, while the rest came from the remaining 23 departments of Peru with a range of 1 to 18 strains per department (Additional file 1: Table S2).

### Phenotypic resistance by APM

The percentages of strains with APM-defined phenotypic resistance included in the study for each one of the drugs were: 86% (427/496) resistant to rifampicin, 94% (464/496) to isoniazid, 45% (218/489) to ethambutol, 31% (155/493) to ethionamide, 11% (56/495) to kanamycin and 11% (54/486) to levofloxacin. In addition, according to the classification of drug resistance profile of the year 2020 [2], 5% (24/496) presented rifampicin mono-resistant TB (RR-TB), 12% (61/496) isoniazid mono-resistant (HR-TB), 76% (376/496) MDR-TB, with a further 5% (27/496) XDR-TB, and 2% (8/496) had different patterns of drug resistance.

### MIC determination using UKMYC6 plate

The microbiological evaluation by the BMD system using the UKMYC6 plate determined that 80% (397/496) presented final growth readings at 14 days and 20% (99/496) at 21 days. Overall, 99% of the readings obtained for each of the drugs showed valid results (MICs inside and outside evaluated ranges). Of these, overall 12% of MIC readings were above the dilution range of the UKMYC6 plate, most frequently rifampicin (68%, 326/480) and rifabutin (46%, 229/494) (Additional file 1: Table S3).

### Comparison between APM and BMD methodology

**Fig. 1:**
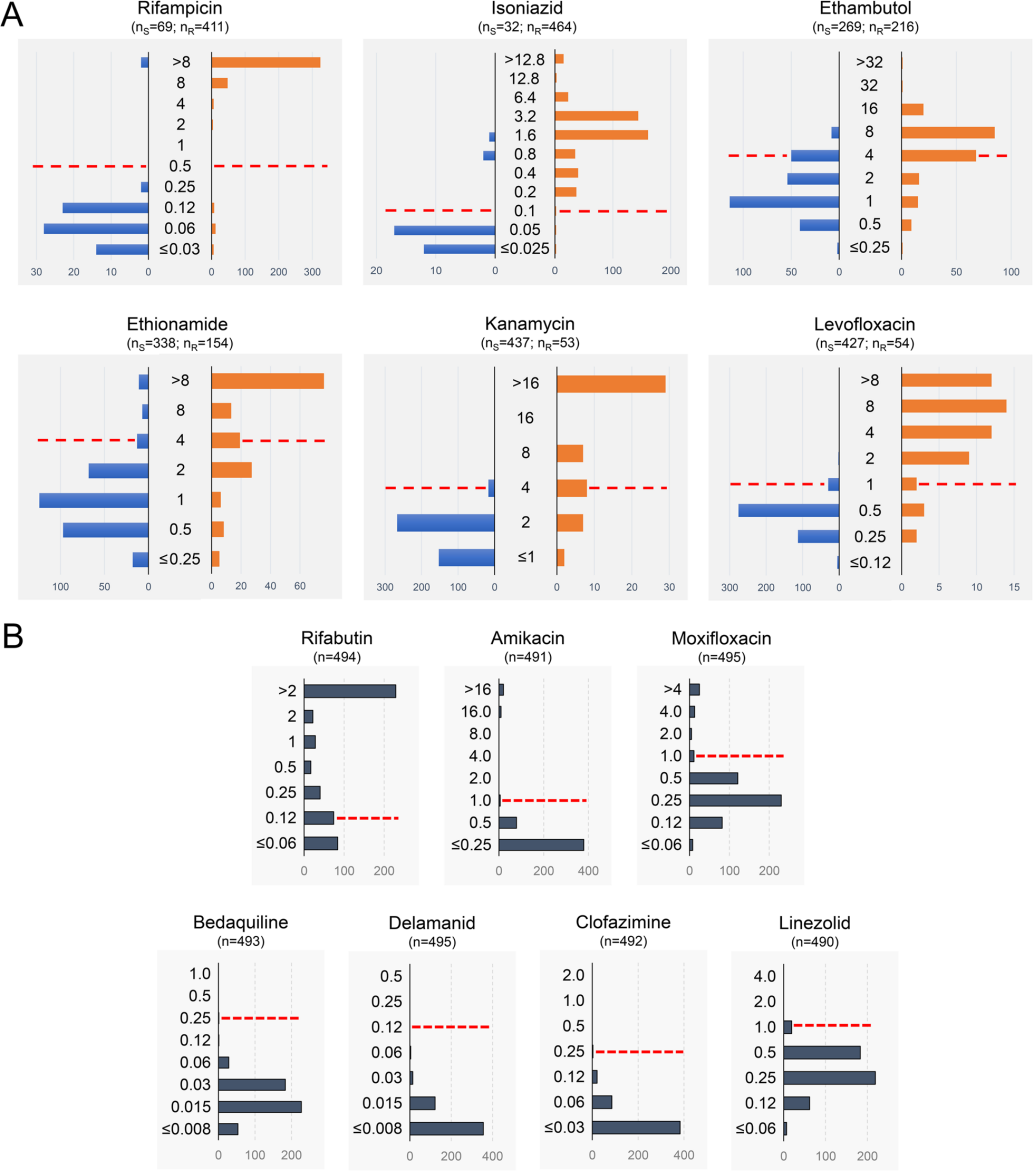
Distribution of MICs in comparison to the results obtained by APM. **A** Analysis obtained from the comparison of susceptibility results by APM, categorized as susceptible (blue bars) or resistant (orange bars), in comparison with the MICs obtained in UKMYC6 plates. **B** Distribution of MICs of drugs analysed only by the BMD plate methodology. For both analyses, the ECOFF/ECVs of the UKMYC6 plates are indicated by dashed lines. The amounts of susceptible (n_S_) and resistant (n_R_) strains by APM are specified for each drug. For the case of drugs that were not evaluated by APM, the total number of strains or measurements (n) performed was specified

Comparative analysis was performed between the APM phenotypic test results and UKMYC6 MICs for 6 drugs: rifampicin, isoniazid, ethambutol, ethionamide, kanamycin and levofloxacin. The phenotypically susceptible or resistant strains by APM were graphed separately by means of histograms showing the MICs values obtained from the UKMYC6 plates (Fig. 1A). The drugs rifampicin, isoniazid, kanamycin, and levofloxacin showed visibly different MIC distributions for APM-susceptible and resistant strains. However, there was considerable overlap of the MIC distribution for strains defined as resistant and susceptible to ethambutol and ethionamide by APM (Fig. 1A; Additional file 1: Fig. S2). For the remaining drugs (rifabutin, amikacin, moxifloxacin, bedaquiline, delamanid, clofazimine, and linezolid) no reference drug susceptibility test (susceptible/resistant) assignment result was available so the MIC distributions for all strains were plotted together (Fig. 1B). The drugs rifabutin, amikacin and moxifloxacin showed the presence of strains with high and low MICs values for which the use of ECOFF/ECVs established the presence of 68% (336/494), 6% (29/491) and 9% (43/495) prevalence of resistant strains, respectively. However, none of the strains presented MICs higher than the ECOFF/ECVs values for the new or repurposed drugs, so they were defined as phenotypically susceptible strains by the BMD plate methodology (Fig. 1B).

For isoniazid, ethambutol, and ethionamide borderline MICs were demonstrated in 16% (77/496), 25% (121/492), and 6% (32/495) of strains, respectively. In the case of isoniazid, all borderline MICs were determined in strains categorized as resistant by APM; while in the cases of ethambutol and ethionamide, borderline MICs were determined in both susceptible and resistant strains (Table 2).

**Table 2:** Phenotypic characterization by APM of results with borderline MICs values

Drug	APM CC (mg/L)	Borderline MIC (mg/L)	APM			Total
			S	R	NA	
Isoniazid	0.2	0.2	0	37	0	77
		0.4	0	40	0	
Ethambutol	5	4	50	68	3	121
Ethionamide	5	4	13	19	0	32

*APM*: 7H10 Agar Proportion Method, *CC*: Critical Concentration, *MIC*: Minimum Inhibitory Concentration, *R*: Number of phenotypically resistant strains, *S*: Number of phenotypically susceptible strains, *NA*: Not Available

### Performance of the BMD plate methodology compared to APM for categorical susceptible/resistant determination

Using the UKMYC6 ECOFF/ECVs values to categorise strains as susceptible or resistant (excluding results with borderline MICs) and comparing these data with APM results available for the six drugs mentioned above resulted in an average categorical agreement of 0.93. The highest categorical concordances were obtained for rifampicin, isoniazid, kanamycin and levofloxacin. Using APM category as the reference, the sensitivity of BMD UKMYC6 plate for detection of drug resistance was highest for isoniazid (0.98) and rifampicin (0.93) and lowest for ethionamide (0.66) and kanamycin (0.68). Specificity was high for all drugs, from 0.91 for isoniazid to 1.0 for kanamycin (Table 3).

**Table 3:** Performance of BMD UKMYC6 plate methodology compared to APM for drug resistance determination

Drug	BMD	APM		Sensitivity (95% CI)	Specificity (95% CI)	PPV (95% CI)	NPV (95% CI)	Categorical agreement	Cohen's kappa
		R	S						
Rifampicin	R	383	2	0.93 (0.90, 0.95)	0.97 (0.90, 1.00)	0.99 (0.98, 1.00)	0.71 (0.60, 0.79)	0.94	0.78
	S	28	67						
Isoniazid	R	381	3	0.98 (0.97, 0.99)	0.91 (0.75, 0.98)	0.99 (0.98, 1.00)	0.83 (0.66, 0.93)	0.98	0.85
	S	6	29						
Ethambutol	R	107	8	0.72 (0.64, 0.79)	0.96 (0.93, 0.98)	0.93 (0.87, 0.97)	0.84 (0.79, 0.88)	0.87	0.71
	S	41	211						
Ethionamide	R	89	18	0.66 (0.57, 0.74)	0.94 (0.91, 0.97)	0.83 (0.75, 0.90)	0.87 (0.83, 0.90)	0.86	0.64
	S	46	307						
Kanamycin	R	36	0	0.68 (0.54, 0.80)	1.00 (0.99, 1.00)	1.00 (0.90, 1.00)	0.96 (0.94, 0.98)	0.97	0.79
	S	17	437						
Levofloxacin	R	47	3	0.87 (0.75, 0.95)	0.99 (0.98, 1.00)	0.94 (0.83, 0.99)	0.98 (0.97, 0.99)	0.98	0.89
	S	7	424						

Results with borderline MICs were excluded from the analysis

*APM*: 7H10 Agar Proportion Method, *BMD*: Broth Microdilution, *R*: Number of phenotypically resistant strains, *S*: Number of phenotypically susceptible strains. *PPV*: Positive Predictive Value, *NPV*: Negative Predictive Value. *95% CI*: 95% Confidence Interval

### WGS used for the analysis of discordant results

Initially, 266 discordant results (present in 191 different strains) were identified for all six drugs tested by the BMD plate method and APM, which included 30 results for rifampicin, 9 for isoniazid, 117 for ethambutol, 83 for ethionamide, 17 for kanamycin, and 10 for levofloxacin (Additional file 1: Table S4). More than 75% (200/266) of the discordant cases were due to results for ethambutol and ethionamide. Subsequent analyses excluding borderline MIC results considerably reduced the number of discordant results. For ethambutol, 68 ‘BMD-susceptible/APM-resistant’ results were excluded, of which 97% (66/68) presented mutations associated with resistance. In contrast, for ethionamide 19 ‘BMD-susceptible/APM-resistant’ discordant results were excluded, of which 47% (9/19) presented mutations associated with resistance. No strain was excluded for the isoniazid results.

Finally, 179 discordant results were obtained (present in 122 different strains). There were no ‘BMD-resistant/APM-susceptible’ discrepancies for kanamycin but for all other agents there were discordant results in both directions. Ethambutol and ethionamide had the highest degrees of disagreement with 13% (49/367) and 14% (64/460), respectively. For most agents the results of genotypic susceptibility testing by WGS gave similar levels support to the results obtained by APM (52%, 93/179) and those obtained by the BMD system (48%, 86/179), with the exception of rifampicin, in which a greater support towards APM results was observed (Table 4).

**Table 4:** Analysis of discordant results between APM and BMD UKMYC6 plate methodology

Drug	Total	APM	BMD	WGS		APM results supported by WGS (%)	BMD results supported by WGS (%)
				**S**	**R**		
Rifampicin	30	S	R	1	1	22 (73)	8 (27)
		R	S	7	21		
Isoniazid	9	S	R	2	1	4 (44)	5 (56)
		R	S	4	2		
Ethambutol	49	S	R	0	8	23 (47)	26 (53)
		R	S	18	23		
Ethionamide	64	S	R	7	11	29 (45)	35 (55)
		R	S	24	22		
Kanamycin	17	S	R	0	0	9 (53)	8 (47)
		R	S	8	9		
Levofloxacin	10	S	R	1	2	6 (60)	4 (40)
		R	S	2	5		

The genotypic result obtained by WGS is shown. Results with borderline MICs were excluded from the analysis

*APM*: 7H10 Agar Proportion Method, *BMD*: Broth Microdilution, *WGS*: Whole Genome Sequencing, *R*: Number of resistant strains, *S*: Number of susceptible strains

## Discussion

This is the first description of the comprehensive drug resistance profile, with MIC distribution of a nationally representative sample of drug-resistant strains of *M. tuberculosis* in Peru. Such analyses are of fundamental importance when considering the local design of standardized treatment regimens for MDR-TB [22]. The study also provided the opportunity to compare indirect drug susceptibility testing (DST) by the proportion method on 7H10 agar against MIC testing using liquid culture by the BMD method.

Overall, there was no resistance to the new and repurposed drugs identified with no strains exceeding the ECOFF/ECV for bedaquiline, delamanid, clofazimine or linezolid. This results agree with previous studies in the Americas region [23] as well as in other contexts [24, 25]. This reflects the sparse usage of these agents within a compassionate use framework prior to their incorporation into national guidelines in 2018 [26] and indicates their introduction into a favourable environment from that timepoint onwards; comparison now with a similarly representative sample of contemporary TB-MDR strains would be instructive and important.

The BMD plate is a convenient tool for the analysis of MICs to any of the drugs used in the treatment of TB [6, 27, 28]. The methodology facilitates addition of new drugs and the range of MICs being tested can be adapted, if necessary, in certain settings [8]. The WHO-recommended critical concentrations for new and repurposed drugs are still provisional [29, 30] and further work to define MIC distributions in a diversity of settings can contribute to refinement [31, 32]. Recently, the WHO has highlighted the great feasibility of the BMD plate methodology for the phenotypic evaluation of various anti-TB drugs, meeting all quality control requirements. Because of this, it has begun to analyze the performance of various BMD systems, including the UKMYC6 plate, in order to provide guidelines for the development of an optimized BMD system that can be recommended for clinical use [33].

The detection of discordant results is a fact that has been documented in previous studies for the different antituberculosis drugs and for both phenotypic and genotypic tests [34], which have been studied in countries with a high burden of drug-resistant strains, including Peru [35]. The fact that the highest percentage of discordant results between the APM and BMD methods have been detected in ethambutol and ethionamide drugs corroborates the existing problem of obtaining inconsistent results for both drugs [36–38]. This was also previously identified as a problem by the WHO, acknowledging that phenotypic DST lacks sufficient reproducibility and is not recommended for these drugs [39]. The considerable decrease in discordant cases through the application of borderline concentrations demonstrates the big challenge of defining a binary susceptible/resistant phenotype for certain drugs. These findings highlight the well-recognised imperfections of all approaches to *M. tuberculosis* DST. Sometimes there is no ‘one right answer’.

The BMD system is presented as an alternative to susceptibility determination against traditional systems such as APM. The latter has longer incubation times and is aimed at only evaluating critical concentrations of a limited number of drugs [39]. Against this, the use of BMD plates allows the simultaneous evaluation of between 12 to 14 drugs including traditional drugs, as well as new and recently repurposed ones [6, 8]. This generates advantages such as: the simultaneous evaluation of a range of concentrations for each drug, a reduction in the times for obtaining results for the complete set of drugs from months to only 14 days, and simplification of workflows in the laboratory [8]. Likewise, the practicality of the design and manufacture allow the BMD system plates to be personalized with different drugs and concentration ranges that best fit the reality of drug resistance prevalence in each country or study [33, 40]. Simultaneous analysis of various anti-tuberculosis drugs would be a great advantage in countries with a high burden of drug resistance such as Peru, where traditional flows establish that a filter is first carried out by means of a susceptibility test against first-line drugs (FLD). Only those with demonstrated FLD resistance are evaluated for susceptibility to second-line agents, including new and repurposed drugs, resulting in further delay in time to results and additional cost. On the other hand, it is estimated that the replacement of drugs and readjustment of concentrations evaluated in microdilution plates would not cause a significant increase in production costs in each country [8].

What is the clinician to make of the information provided by the laboratory and how should the laboratory present it? There is a reluctance to share MIC data with clinicians who lack the training to interpret it. Few laboratory scientists and even fewer clinicians understand the complexities of the pharmacokinetics of TB drugs and how this relates to the MIC for a particular drug for a particular strain, so it still seems reasonable to try to simplify the message to the binary susceptible/resistant call where possible.

The value of MIC data lies in understanding the drift in the distribution in well characterised populations over time (public health usage) and in case management when therapeutic options are very limited but dosage increases might facilitate efficacy (clinical usage) [41]. Periodically re-evaluating the national MIC distribution profile, in particular for the new and repurposed drugs which have been introduced since this strain sample was obtained, would shed important light upon the speed at which drug-resistant strains are (or are not) emerging, information which might not be immediately apparent from simply looking at the binary susceptible/resistant data.

An important strength of this analysis is the national representativity. All strains identified nationally during the study period should have been sent to the National Mycobacteria Reference Laboratory for further testing so stratified sampling of the strain bank according to MDR-TB burden during the study period ensured a comprehensive and proportionate national coverage. The availability and use of WGS for discrepant analysis was a critically important enhancement that reinforced the importance of not depending upon a single methodology as the ‘gold standard’. A limitation of the analysis was the lack of APM data for the new and repurposed agents, reflecting the earlier time period during which the original proportion method testing was done, and highlighting the power and versatility of the BMD plate methodology in accommodating a large number of drugs within a single assay.

The rapid expansion of the use of WGS for TB DST [42, 43], the growing library of identified resistance-conferring SNPs for all drugs and the tumbling cost (sequencing of the MTB genome is now no more expensive than MGIT phenotypic DST for 4 agents in the UK), place WGS as a likely near-horizon successor to phenotypic DST in settings where the infrastructure allows. Web-based tools for WGS interpretation can deliver (almost instantaneously) a ‘resistance probability’ for every drug based upon SNP identification in an uploaded sequence. This is derived by comparison with a large iterative database of paired phenotypic-genotypic data combined with some prediction modelling. Crucially for the clinician, the ‘probability’ acknowledges the uncertainty inherent in the result, allowing for a more intelligent and informed decision-making.

## Conclusion

The susceptibility determination system by the BMD method using the UKMYC6 plate allows the complete susceptibility characterization, through the determination of MICs, of drug-resistant *Mycobacterium tuberculosis* strains in Peru. This methodology shows good diagnostic performance for rifampicin, isoniazid, ethambutol, ethionamide, kanamycin and levofloxacin. It also allows the characterization of MICs for additional first and second-line drugs as well as for new and repurposed drugs.

## Supplementary Information

**Additional file 1: Supplementary Method:** DNA extraction procedure. **Fig. S1:** UKMYC6 microdilution plate design and range of concentrations. **Fig. S2: **Simplified histogram of MIC distribution and APM results for compared drugs. **Table S1:** Ternary categorization system based on MICs obtained by the Broth microdilution UKMYC6 plate. **Table S2:** Geographical distribution of the MTB Peruvian strains included in the study. **Table S3:** Summary and classification of MIC readings obtained in the BMD UKMYC6 plate methodology. **Table S4:** Discordant results between APM and BMD UKMYC6 plate methodology.

## Data Availability

The raw sequence data (fastq.gz files) are available from the European Nucleotide Archive (ENA) under the project accession number: PRJEB41199 (individual accession codes are listed in Additional file 1: Table S4).

## Abbreviations

APM: 7H10 Agar Proportion Method
BMD: Broth Microdilution
CC: Critical Concentration
CFU: Colony-Forming Unit
CLSI: Clinical and Laboratory Standards Institute
CRyPTIC: Comprehensive Resistance Prediction for Tuberculosis: an International Consortium
DST: Drug Susceptibility Test
ECOFF/ECV: Epidemiological Cut-off Value
ENA: European Nucleotide Archive
INS: Instituto Nacional de Salud
LRNM: Laboratorio de Referencia Nacional de Micobacterias
LSHTM: The London School of Hygiene & Tropical Medicine
MDR-TB: Multidrug-resistant tuberculosis
MIC: Minimum Inhibitory Concentration
MTB: *Mycobacterium tuberculosis*
OADC: Oleic acid Albumin Dextrose Catalase
SNP: Single Nucleotide Polymorphism
TB: Tuberculosis
UPCH: Universidad Peruana Cayetano Heredia
WGS: Whole Genome Sequencing
WHO: World Health Organization
XDR-TB: Extensively drug-resistant tuberculosis
