# Supplementary material for: Evaluation of the broth microdilution plate methodology for susceptibility testing of Mycobacterium tuberculosis in Peru
Source: BMC Infect Dis. 2022 Aug 24;22:705. doi: 10.1186/s12879-022-07677-9 (PMC9399989; doi:10.1186/s12879-022-07677-9)
Supplement: Supplementary file 1 — Additional file 1: Supplementary Method: DNA extraction procedure. Fig. S1: UKMYC6 microdilution plate design and range of concentrations. Fig. S2: Simplified histogram of MIC distribution and APM results for compared drugs. Table S1: Ternary categorization system based on MICs obtained by the Broth microdilution UKMYC6 plate. Table S2: Geographical distribution of the MTB Peruvian strains included in the study. Table S3: Summary and classification of MIC readings obtained in the BMD UKMYC6 plate methodology. Table S4: Discordant results between APM and BMD UKMYC6 plate methodology. [file 12879_2022_7677_MOESM1_ESM.doc]

**SUPPLEMENTARY DATA**

**Supplementary Method:** DNA extraction procedure from *Mycobacterium tuberculosis* strains using phenol-chloroform method.

Using a sterile loop, harvest several colonies of mycobacteria and diluted in 500 µL of TE buffer.

Heat to 80 °C for 20 minutes.

Add 50 µL of lysozyme (10 mg/mL), vortex and incubate overnight at
37 °C.

Add 75 µL of 10% SDS and 20 µL of Proteinase K (20 mg/mL), vortex and incubate at 65 °C for at least 3 hours. And vortex for 5 seconds every 20 minutes

Add 100 µL of 5M NaCl and 100 µL of CTAB/NaCl preheated to 65 °C, vortex and incubate at 65 °C for 10 minutes.

Add 750 µL of phenol-chloroform-isoamyl alcohol (25:24:1), vortex and centrifuge for 5 minutes at 10,000 rpm.

Obtain the supernatant and transfer it to another tube.

Add 750 µL of chloroform-isoamyl alcohol (24:1), vortex and centrifuge for 5 minutes at 10,000 rpm.

Obtain the supernatant and transfer it to another tube.

Add 1 mL of absolute alcohol cold and frozen at -70 ° C for 15 minutes.

Centrifuge for 8 minutes at 10,000 rpm. Discard the liquid and dry the pellet.

Add 1 mL of 70% cold alcohol and centrifuge for 3 minutes at 10,000 rpm. Discard the liquid and dry the pellet.

Dilute 100 µL DNA in TE buffer (10 mM Tris-HCl, 1 mM EDTA).

**Figure S1:** UKMYC6 microdilution plate design and range of concentrations used for each of the 13 drugs tested.

**Figure S2:** Simplified histogram of Minimum Inhibitory Concentrations (MIC) distribution and 7H10 Agar Proportion Method (APM) results for the six drugs compared. APM results are categorized as susceptible (blue bars) or resistant (orange bars), Critical concentrations are established for APM (yellow wedge) and broth macrodilution BACTEC/MGIT (light blue wedge) systems. Also, CRyPTIC proposed Epidemiological Cut-off Values for UKMYC6 microdilution plate (red wedge) are specified.


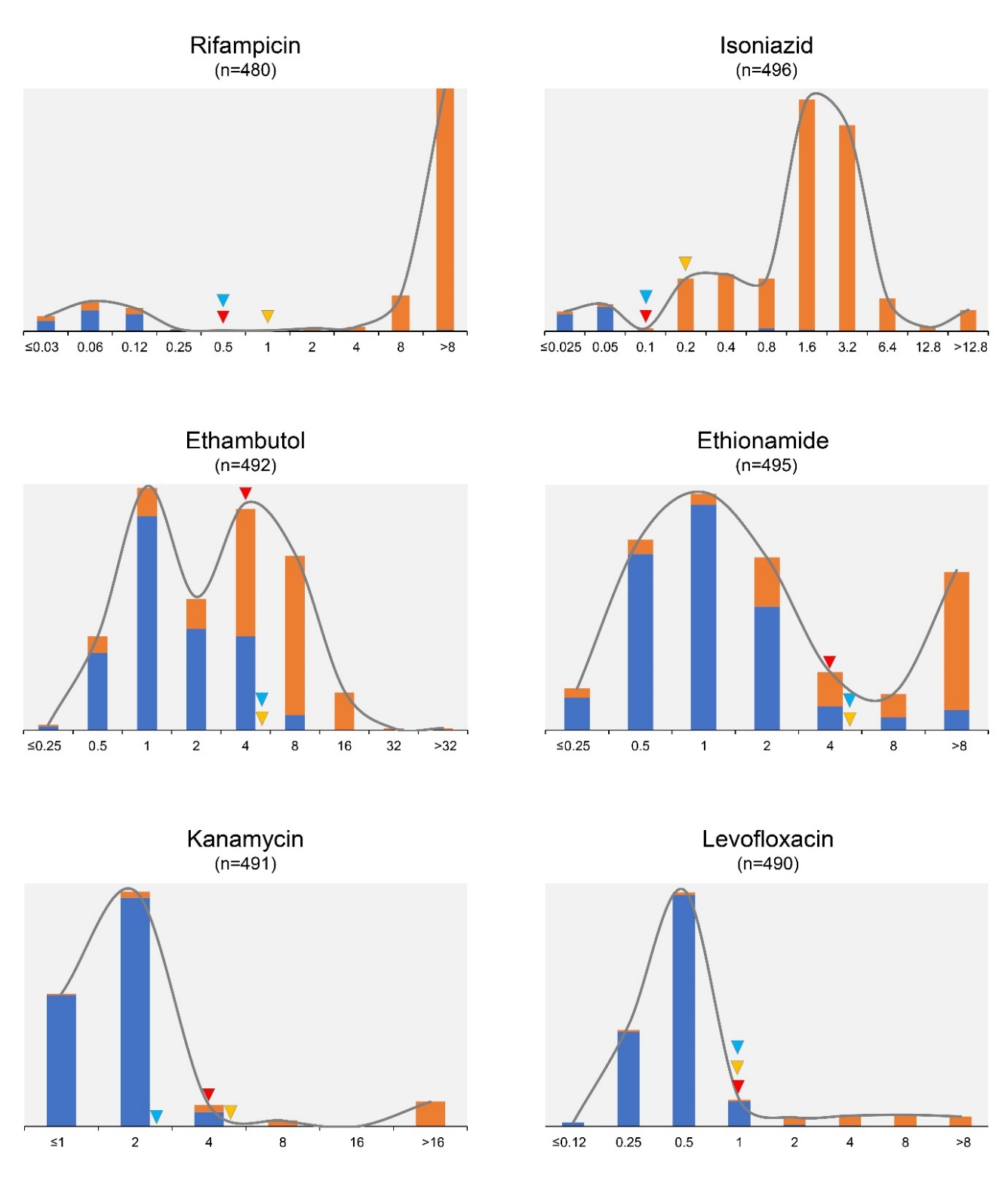


**Table S1:** Ternary categorization system based on MICs obtained by the Broth microdilution UKMYC6 plate.

| **Drug** | **UKMYC6 ECOFF/ECV (mg/L)** | **Susceptible (mg/L)** | **Intermediate (mg/L)** | **Resistant (mg/L)** |  |
| --- | --- | --- | --- | --- | --- |
|  |  |  |  |  |  |
| Rifampicin | 0.5 | ≤ 0.5 | - | ≥ 1 |  |
| Rifabutin | 0.12 | ≤ 0.12 | - | ≥ 0.25 |  |
| Isoniazid | 0.1 | ≤ 0.1 | 0.2, 0.4 | ≥ 0.8 |  |
| Ethambutol | 4.0 | ≤ 2 | 4 | ≥ 8 |  |
| Moxifloxacin | 1.0 | ≤ 1 | - | ≥ 2 |  |
| Levofloxacin | 1.0 | ≤ 1 | - | ≥ 2 |  |
| Kanamycin | 4.0 | ≤ 4 | - | ≥ 8 |  |
| Amikacin | 1.0 | ≤ 1 | - | ≥ 2 |  |
| Ethionamide | 4.0 | ≤ 2 | 4 | ≥ 8 |  |
| Linezolid | 1.0 | ≤ 1 | - | ≥ 2 |  |
| Clofazimine | 0.25 | ≤ 0.25 | - | ≥ 0.5 |  |
| Bedaquiline | 0.25 | ≤ 0.25 | - | ≥ 0.5 |  |
| Delamanid | 0.12 | ≤ 0.12 | - | ≥ 0.25 |  |

**ECOFF/ECV**: Epidemiological Cut-off Values previously established by the CRyPTIC consortium.

**Table S2:** Geographical distribution of the *Mycobacterium tuberculosis* Peruvian strains included in the study.

| **Location (Department)** | **N** | **%** |
| --- | --- | --- |
| Piura | 6 | 1.2 |
| Lambayeque | 5 | 1.0 |
| La Libertad | 18 | 3.6 |
| Cajamarca | 3 | 0.6 |
| Amazonas | 2 | 0.4 |
| Tumbes | 2 | 0.4 |
| San Martín | 5 | 1.0 |
| Loreto | 5 | 1.0 |
| Lima and Callao | 347 | 70.0 |
| Ica | 13 | 2.6 |
| Ancash | 12 | 2.4 |
| Huánuco | 8 | 1.6 |
| Pasco | 1 | 0.2 |
| Huancavelica | 2 | 0.4 |
| Junín | 10 | 2.0 |
| Ucayali | 15 | 3.0 |
| Moquegua | 5 | 1.0 |
| Tacna | 5 | 1.0 |
| Ayacucho | 3 | 0.6 |
| Apurímac | 5 | 1.0 |
| Arequipa | 6 | 1.2 |
| Cusco | 6 | 1.2 |
| Puno | 5 | 1.0 |
| Madre de Dios | 7 | 1.4 |
| Total | 496 | 100 |

**Table S3:** Summary and classification of MIC readings obtained in the BMD UKMYC6 plate methodology.

| **Drug** | **MICs outside range ^a^** | **MICs inside range** | **Contaminated wells** | **Skipped wells** | **Dry wells** | **N° Valid results ^b^ (%)** |  |
| --- | --- | --- | --- | --- | --- | --- | --- |
|  |  |  |  |  |  |  |  |
| Rifampicin | 326 | 154 | 0 | 16 | 0 | 480 (96.8) |  |
| Rifabutin | 229 | 265 | 2 | 0 | 0 | 494 (99.6) |  |
| Isoniazid | 15 | 481 | 0 | 0 | 0 | 496 (100.0) |  |
| Ethambutol | 1 | 491 | 4 | 0 | 0 | 492 (99.2) |  |
| Moxifloxacin | 25 | 470 | 1 | 0 | 0 | 495 (99.8) |  |
| Levofloxacin | 12 | 478 | 6 | 0 | 0 | 490 (98.8) |  |
| Kanamycin | 29 | 462 | 0 | 5 | 0 | 491 (99.0) |  |
| Amikacin | 20 | 471 | 3 | 2 | 0 | 491 (99.0) |  |
| Ethionamide | 88 | 407 | 1 | 0 | 0 | 495 (99.8) |  |
| Linezolid | 0 | 490 | 6 | 0 | 0 | 490 (98.8) |  |
| Clofazimine | 0 | 492 | 4 | 0 | 0 | 492 (99.2) |  |
| Bedaquiline | 0 | 493 | 3 | 0 | 0 | 493 (99.4) |  |
| Delamanid | 0 | 495 | 1 | 0 | 0 | 495 (99.8) |  |
| Total | 745 | 5649 | 31 | 23 | 0 |  |  |

^a^ MICs were above the maximum concentration evaluated.

^b^ Valid results = MICs outside range + MICs inside range.

**Table S4**: Discordant results between APM and BMD UKMYC6 plate methodology. ENA accession codes and genotypic (through whole genome sequencing) results are specified (n=266).

| **Drug** | **ENA code** | **Phenotypic/genotypic results** | | | |
| --- | --- | --- | --- | --- | --- |
|  |  |  |  |  |  |
| **Rifampicin** (n=30) |  | **MIC** (mg/L) | **BMD**  (ECV = 0.5 mg/L) | **APM**  (CC = 1.0 mg/L) | **WGS** |
|  | ERR4812806 | 0.03 | S | R | R |
|  | ERR4812814 | 0.06 | S | R | R |
|  | ERR4812834 | 0.12 | S | R | S |
|  | ERR4812875 | 0.12 | S | R | R |
|  | ERR4812877 | 0.12 | S | R | R |
|  | ERR4813297 | 0.12 | S | R | R |
|  | ERR4813316 | 0.03 | S | R | R |
|  | ERR4813317 | 0.06 | S | R | S |
|  | ERR4813407 | 0.06 | S | R | R |
|  | ERR4813424 | >8 | R | S | S |
|  | ERR4813445 | 0.12 | S | R | R |
|  | ERR4813465 | 0.03 | S | R | S |
|  | ERR4813467 | 0.5 | S | R | R |
|  | ERR4813035 | 0.25 | S | R | S |
|  | ERR4813059 | 0.06 | S | R | R |
|  | ERR4813060 | 0.06 | S | R | R |
|  | ERR4813069 | 0.12 | S | R | R |
|  | ERR4813658 | 0.06 | S | R | S |
|  | ERR4812981 | 0.12 | S | R | R |
|  | ERR4813019 | 0.03 | S | R | R |
|  | ERR4813695 | 0.06 | S | R | R |
|  | ERR4813688 | 0.06 | S | R | R |
|  | ERR4813585 | 0.03 | S | R | S |
|  | ERR4813663 | 0.06 | S | R | R |
|  | ERR4813627 | 0.12 | S | R | R |
|  | ERR4813673 | 0.03 | S | R | S |
|  | ERR4813675 | 0.06 | S | R | R |
|  | ERR4813626 | >8 | R | S | R |
|  | ERR4813646 | 0.06 | S | R | R |
|  | ERR4813714 | 0.06 | S | R | R |
|  |  |  |  |  |  |
| **Isoniazid** (n=9) |  | **MIC** (mg/L) | **BMD** (ECV = 0.1 mg/L) | **APM** (CC = 0.2 mg/L) | **WGS** |
|  | ERR4812834 | 0.025 | S | R | S |
|  | ERR4812873 | 0.8 | R | S | R |
|  | ERR4812876 | 1.6 | R | S | S |
|  | ERR4812880 | 0.1 | S | R | R |
|  | ERR4813317 | 0.05 | S | R | S |
|  | ERR4813424 | 0.8 | R | S | S |
|  | ERR4813658 | 0.05 | S | R | S |
|  | ERR4813585 | 0.025 | S | R | S |
|  | ERR4813663 | 0.1 | S | R | R |
|  |  |  |  |  |  |
| **Ethambutol** (n=117) |  | **MIC** (mg/L) | **BMD** (ECV = 4.0 mg/L) | **APM** (CC = 5.0 mg/L) | **WGS** |
|  | ERR4812849 | 4 | S | R | R |
|  | ERR4812803 | 4 | S | R | R |
|  | ERR4812839 | 4 | S | R | S |
|  | ERR4812949 | 4 | S | R | R |
|  | ERR4812812 | 1 | S | R | S |
|  | ERR4812951 | 8 | R | S | R |
|  | ERR4812835 | 0.5 | S | R | R |
|  | ERR4812834 | 1 | S | R | S |
|  | ERR4812859 | 4 | S | R | R |
|  | ERR4812838 | 0.5 | S | R | R |
|  | ERR4812818 | 4 | S | R | R |
|  | ERR4812816 | 4 | S | R | R |
|  | ERR4812805 | 4 | S | R | R |
|  | ERR4812873 | 2 | S | R | S |
|  | ERR4812875 | 1 | S | R | R |
|  | ERR4812876 | 1 | S | R | S |
|  | ERR4812877 | 1 | S | R | R |
|  | ERR4812878 | 4 | S | R | R |
|  | ERR4813297 | 1 | S | R | R |
|  | ERR4813299 | 2 | S | R | S |
|  | ERR4813315 | 4 | S | R | R |
|  | ERR4813330 | 8 | R | S | R |
|  | ERR4813337 | 4 | S | R | R |
|  | ERR4813349 | 4 | S | R | R |
|  | ERR4813351 | 4 | S | R | S |
|  | ERR4813360 | 4 | S | R | R |
|  | ERR4813367 | 4 | S | R | R |
|  | ERR4813368 | 2 | S | R | S |
|  | ERR4813372 | 4 | S | R | R |
|  | ERR4813373 | 1 | S | R | S |
|  | ERR4813381 | 2 | S | R | R |
|  | ERR4813385 | 4 | S | R | R |
|  | ERR4813386 | 4 | S | R | R |
|  | ERR4813389 | 1 | S | R | S |
|  | ERR4813398 | 4 | S | R | R |
|  | ERR4813402 | 4 | S | R | R |
|  | ERR4813406 | 2 | S | R | S |
|  | ERR4813407 | 0.5 | S | R | R |
|  | ERR4813416 | 2 | S | R | S |
|  | ERR4813417 | 4 | S | R | R |
|  | ERR4813419 | 4 | S | R | R |
|  | ERR4813420 | 4 | S | R | R |
|  | ERR4813427 | 8 | R | S | R |
|  | ERR4813432 | 4 | S | R | R |
|  | ERR4813435 | 4 | S | R | R |
|  | ERR4813451 | 4 | S | R | R |
|  | ERR4813453 | 4 | S | R | R |
|  | ERR4813454 | 4 | S | R | R |
|  | ERR4813456 | 4 | S | R | R |
|  | ERR4813459 | 4 | S | R | R |
|  | ERR4813461 | 8 | R | S | R |
|  | ERR4813465 | 4 | S | R | R |
|  | ERR4813466 | 2 | S | R | S |
|  | ERR4813470 | 4 | S | R | R |
|  | ERR4813021 | 2 | S | R | R |
|  | ERR4813601 | 4 | S | R | R |
|  | ERR4812883 | 4 | S | R | R |
|  | ERR4812889 | 4 | S | R | R |
|  | ERR4812897 | 4 | S | R | R |
|  | ERR4813035 | 2 | S | R | S |
|  | ERR4813042 | 4 | S | R | R |
|  | ERR4813048 | 4 | S | R | R |
|  | ERR4813049 | 1 | S | R | S |
|  | ERR4813052 | 4 | S | R | R |
|  | ERR4813054 | 4 | S | R | R |
|  | ERR4813064 | 4 | S | R | R |
|  | ERR4813069 | 0.5 | S | R | R |
|  | ERR4813078 | 2 | S | R | R |
|  | ERR4812963 | 0.5 | S | R | R |
|  | ERR4812972 | 4 | S | R | R |
|  | ERR4813658 | 2 | S | R | S |
|  | ERR4812978 | 2 | S | R | R |
|  | ERR4812981 | 2 | S | R | R |
|  | ERR4812984 | 4 | S | R | R |
|  | ERR4812989 | 4 | S | R | R |
|  | ERR4812992 | 4 | S | R | R |
|  | ERR4813002 | 4 | S | R | R |
|  | ERR4813003 | 2 | S | R | R |
|  | ERR4813722 | 4 | S | R | R |
|  | ERR4813007 | 4 | S | R | R |
|  | ERR4813014 | 4 | S | R | R |
|  | ERR4813070 | 4 | S | R | R |
|  | ERR4813075 | 4 | S | R | R |
|  | ERR4813076 | 4 | S | R | R |
|  | ERR4813079 | 4 | S | R | R |
|  | ERR4813082 | 4 | S | R | R |
|  | ERR4813090 | 8 | R | S | R |
|  | ERR4813091 | 4 | S | R | R |
|  | ERR4813092 | 2 | S | R | S |
|  | ERR4812947 | 8 | R | S | R |
|  | ERR4812960 | 0.5 | S | R | S |
|  | ERR4813011 | 1 | S | R | R |
|  | ERR4813019 | 0.5 | S | R | R |
|  | ERR4813695 | 0.25 | S | R | R |
|  | ERR4813688 | 1 | S | R | R |
|  | ERR4813616 | 4 | S | R | R |
|  | ERR4813660 | 4 | S | R | R |
|  | ERR4813671 | 4 | S | R | R |
|  | ERR4813726 | 4 | S | R | R |
|  | ERR4813727 | 4 | S | R | R |
|  | ERR4813654 | 4 | S | R | R |
|  | ERR4813689 | 8 | R | S | R |
|  | ERR4813627 | 0.5 | S | R | R |
|  | ERR4813673 | 0.5 | S | R | S |
|  | ERR4813706 | 4 | S | R | R |
|  | ERR4813709 | 4 | S | R | R |
|  | ERR4813711 | 4 | S | R | R |
|  | ERR4813604 | 2 | S | R | R |
|  | ERR4813719 | 8 | R | S | R |
|  | ERR4813612 | 4 | S | R | R |
|  | ERR4813615 | 4 | S | R | R |
|  | ERR4813729 | 1 | S | R | S |
|  | ERR4813603 | 4 | S | R | R |
|  | ERR4813696 | 4 | S | R | R |
|  | ERR4813646 | 1 | S | R | R |
|  | ERR4813685 | 1 | S | R | R |
|  | ERR4813714 | 1 | S | R | R |
|  |  |  |  |  |  |
| **Ethionamide** (n=83) |  | **MIC** (mg/L) | **BMD** (ECV = 4.0 mg/L) | **APM** (CC = 5.0 mg/L) | **WGS** |
|  | ERR4812812 | 1 | S | R | S |
|  | ERR4812835 | 0.25 | S | R | R |
|  | ERR4812814 | 0.25 | S | R | R |
|  | ERR4812816 | 2 | S | R | S |
|  | ERR4812875 | 0.5 | S | R | S |
|  | ERR4812878 | 2 | S | R | R |
|  | ERR4813294 | 0.25 | S | R | R |
|  | ERR4813308 | 2 | S | R | S |
|  | ERR4813313 | >8 | R | S | R |
|  | ERR4813316 | 0.25 | S | R | R |
|  | ERR4813325 | 8 | R | S | S |
|  | ERR4813333 | 4 | S | R | R |
|  | ERR4813349 | 2 | S | R | R |
|  | ERR4813354 | 1 | S | R | S |
|  | ERR4813358 | 2 | S | R | R |
|  | ERR4813608 | 2 | S | R | R |
|  | ERR4813368 | 2 | S | R | S |
|  | ERR4813374 | 2 | S | R | S |
|  | ERR4813375 | >8 | R | S | R |
|  | ERR4813384 | 1 | S | R | S |
|  | ERR4813385 | 2 | S | R | R |
|  | ERR4813386 | 4 | S | R | S |
|  | ERR4813394 | 4 | S | R | R |
|  | ERR4813398 | 4 | S | R | S |
|  | ERR4813402 | 2 | S | R | R |
|  | ERR4813405 | >8 | R | S | S |
|  | ERR4813407 | 0.5 | S | R | R |
|  | ERR4813411 | >8 | R | S | R |
|  | ERR4813413 | 1 | S | R | S |
|  | ERR4813417 | 4 | S | R | S |
|  | ERR4813424 | 2 | S | R | S |
|  | ERR4813429 | 4 | S | R | S |
|  | ERR4813434 | >8 | R | S | R |
|  | ERR4813445 | 8 | R | S | R |
|  | ERR4813449 | 2 | S | R | R |
|  | ERR4813465 | 4 | S | R | S |
|  | ERR4813466 | >8 | R | S | S |
|  | ERR4813472 | >8 | R | S | S |
|  | ERR4812887 | 4 | S | R | S |
|  | ERR4812894 | 2 | S | R | R |
|  | ERR4812900 | 8 | R | S | S |
|  | ERR4813026 | 4 | S | R | S |
|  | ERR4813028 | >8 | R | S | R |
|  | ERR4813040 | >8 | R | S | R |
|  | ERR4813042 | 4 | S | R | S |
|  | ERR4813059 | 0.5 | S | R | R |
|  | ERR4813060 | 0.25 | S | R | S |
|  | ERR4813068 | 2 | S | R | R |
|  | ERR4813078 | 2 | S | R | S |
|  | ERR4812978 | 0.5 | S | R | S |
|  | ERR4812984 | 2 | S | R | S |
|  | ERR4812989 | 2 | S | R | R |
|  | ERR4812991 | 0.5 | S | R | S |
|  | ERR4812995 | 2 | S | R | S |
|  | ERR4812998 | 2 | S | R | R |
|  | ERR4813001 | 2 | S | R | R |
|  | ERR4813003 | 8 | R | S | S |
|  | ERR4813010 | >8 | R | S | R |
|  | ERR4813640 | >8 | R | S | R |
|  | ERR4813083 | 4 | S | R | S |
|  | ERR4812947 | 2 | S | R | S |
|  | ERR4812988 | 2 | S | R | S |
|  | ERR4813011 | 1 | S | R | S |
|  | ERR4813622 | 8 | R | S | R |
|  | ERR4813620 | 4 | S | R | R |
|  | ERR4813648 | 4 | S | R | R |
|  | ERR4813585 | 0.5 | S | R | S |
|  | ERR4813624 | 4 | S | R | R |
|  | ERR4813654 | 2 | S | R | S |
|  | ERR4813659 | 8 | R | S | R |
|  | ERR4813662 | 1 | S | R | R |
|  | ERR4813680 | 4 | S | R | R |
|  | ERR4813689 | 0.5 | S | R | S |
|  | ERR4813610 | 0.5 | S | R | S |
|  | ERR4813591 | 8 | R | S | S |
|  | ERR4813604 | 4 | S | R | R |
|  | ERR4813694 | 2 | S | R | R |
|  | ERR4813715 | 2 | S | R | R |
|  | ERR4813734 | 2 | S | R | R |
|  | ERR4813735 | 4 | S | R | S |
|  | ERR4813657 | 2 | S | R | S |
|  | ERR4813672 | 4 | S | R | R |
|  | ERR4813676 | 4 | S | R | R |
|  |  |  |  |  |  |
| **Kanamycin** (n=17) |  | **MIC** (mg/L) | **BMD** (ECV = 4.0 mg/L) | **APM** (CC = 5.0 mg/L) | **WGS** |
|  | ERR4812811 | 1 | S | R | S |
|  | ERR4812825 | 4 | S | R | R |
|  | ERR4812835 | 2 | S | R | R |
|  | ERR4813316 | 2 | S | R | R |
|  | ERR4813357 | 4 | S | R | S |
|  | ERR4813385 | 4 | S | R | R |
|  | ERR4813407 | 2 | S | R | R |
|  | ERR4813473 | 2 | S | R | S |
|  | ERR4813475 | 4 | S | R | S |
|  | ERR4812977 | 4 | S | R | S |
|  | ERR4813658 | 2 | S | R | S |
|  | ERR4812978 | 2 | S | R | R |
|  | ERR4812987 | 4 | S | R | R |
|  | ERR4812988 | 2 | S | R | S |
|  | ERR4813673 | 1 | S | R | S |
|  | ERR4813715 | 4 | S | R | R |
|  | ERR4813734 | 4 | S | R | R |
|  |  |  |  |  |  |
| **Levofloxacin** (n=10) |  | **MIC** (mg/L) | **BMD** (ECV = 1.0 mg/L) | **APM** (CC = 1.0 mg/L) | **WGS** |
|  | ERR4813405 | 4 | R | S | R |
|  | ERR4813417 | 2 | R | S | S |
|  | ERR4813447 | 1 | S | R | S |
|  | ERR4813658 | 0.5 | S | R | S |
|  | ERR4812978 | 0.5 | S | R | R |
|  | ERR4812974 | 2 | R | S | R |
|  | ERR4813673 | 0.25 | S | R | R |
|  | ERR4813706 | 1 | S | R | R |
|  | ERR4813709 | 0.25 | S | R | R |
|  | ERR4813647 | 0.5 | S | R | R |

**APM**: 7H10 Agar Proportion Method. **BMD**: Broth microdilution. **WGS**: Whole Genome Sequencing. **ENA**: European Nucleotide Archive. **ECV**: Epidemiological Cut-off Values. **CC**: Critical concentration. **R**: Number of resistant strains. **S**: Number of susceptible strains.
